# Association of Catheter Ablation and Reduced Incidence of Dementia among Patients with Atrial Fibrillation during Long-Term Follow-Up: A Systematic Review and Meta-Analysis of Observational Studies

**DOI:** 10.3390/jcdd9050140

**Published:** 2022-04-30

**Authors:** Andrea Saglietto, Andrea Ballatore, Henri Xhakupi, Gaetano Maria De Ferrari, Matteo Anselmino

**Affiliations:** Division of Cardiology, “Città della Salute e della Scienza di Torino” Hospital, Department of Medical Sciences, University of Turin, 10126 Torino, Italy; andrea.saglietto@live.com (A.S.); andrea.ballatore3@gmail.com (A.B.); henri.xhakupi@edu.unito.it (H.X.); matteo.anselmino@unito.it (M.A.)

**Keywords:** atrial fibrillation, atrial fibrillation catheter ablation, dementia, cognitive decline

## Abstract

Background: Atrial fibrillation (AF) is independently associated with the onset of cognitive decline/dementia. AF catheter ablation (AFCA) is the most effective treatment strategy in terms of sinus rhythm maintenance, but its effects on dementia prevention remain under investigation. The aim of the present study was to perform a systematic review and meta-analysis of the presently available studies exploring the effect of AFCA on dementia occurrence. Methods: PubMed/MEDLINE databases were screened for articles through 14 March 2022 reporting adjusted time-to-event outcome data comparing AFCA and non-AFCA cohorts in terms of de novo dementia occurrence. A random effect meta-analysis was performed to estimate the meta-analytic hazard ratio (HR) of dementia occurrence in AFCA vs. non-AFCA cohorts, as well as the meta-analytic incidence rate of dementia in the non-AFCA cohort. Based on the aforementioned estimates, the number needed to treat (NNT), projected at median follow-up, was derived. Results: Four observational studies were included in the analysis, encompassing 40,146 patients (11,312 in the AFCA cohort; 28,834 in the non-AFCA cohort). AFCA conferred a significant protection to the development of dementia with an overall HR of 0.52 (95% CI 0.35–0.76). The incidence rate of dementia in the non-AFCA group was 1.12 events per 100 person-year (95% CI 0.47–2.67). The derived NNT projected to the median follow-up (4.5 years) was 41. Conclusion: AFCA is associated with a nearly 50% reduction in dementia occurrence during a median 4.5-year follow-up. Future randomized clinical trials are needed to reinforce these findings.

## 1. Introduction

Atrial fibrillation (AF), the most frequent cardiac arrhythmia in adults, is expected to rise in prevalence in the coming decades [1,2]. Similarly, the dementia burden is predicted to increase more than twofold by 2050, possibly affecting more than 100 million patients worldwide [3]. These two conditions share many common risk factors, such as ageing, hypertension, diabetes and heart failure, and, in addition, a direct cause and effect relationship, independently relating incident AF to cognitive decline/dementia, has recently emerged [4]. In fact, the effects of arrhythmia are not limited to clinical cerebrovascular accidents (CVAs); there are various mechanisms which might explain an accelerated cognitive decline and the eventual onset of dementia in patients suffering AF: hemodynamic mechanisms, subclinical microembolic cerebral infarctions, subclinical cerebral microbleeds and impaired cerebrovascular reactivity [5,6,7,8,9,10].

Among the possible therapeutic strategies for AF management, catheter ablation (AFCA) is the most effective in achieving rhythm control and improving quality of life [11,12,13]. Lately, the possible role of rhythm control strategies in preventing cognitive decline has been investigated [14,15,16,17,18,19]; however, the effect of AFCA on dementia occurrence, a highly relevant medical and socio-economical event, has rarely been studied and remains matter of debate.

Given these premises, the aim of the present study, as along with providing an updated overview on the topic, was to perform a systematic review and meta-analysis of the studies exploring the effect of AFCA on dementia incidence to assess quantitative estimates of the potential benefit.

## 2. Methods

### 2.1. Search Strategy and Primary Outcome

The analysis was performed according to the PRISMA checklist for transparency [20] (Supplementary Table S1). PubMed/MEDLINE and EMBASE databases were screened through 14 March 2022, using the following search strategy: ((atrial fibrillation OR AF OR afib) AND (PCA OR ablation OR catheter ablation OR AFCA OR catheter)) AND (dementia OR cogn *) NOT (conference abstract).

Randomized controlled trials, prospective and retrospective observational studies were considered eligible if:

- They compared de novo dementia occurrence in AFCA vs. non-AFCA (medical) cohorts, providing adjusted estimates based on matching and/or multivariable analysis statistical techniques; and
- They reported time-to-event risk estimate for the outcome (i.e., hazard ratio, HR).

Non-English language studies, abstracts, editorials, letters, systematic reviews, meta-analysis and unpublished data were excluded. Two investigators (A.B. and H.X.) independently reviewed the titles/abstracts and studies to determine their eligibility based on the inclusion criteria and extracted all relevant data (i.e., population’s baseline characteristics, hazard ratio for dementia, dementia incidence). A third investigator (A.S.) resolved disagreement. Quality assessment of the included studies was performed using the Newcastle–Ottawa Scale for observational studies. The primary outcome of the analysis was dementia incidence in patients who had undergone AFCA compared to those managed by drug treatment.

### 2.2. Statistical Analysis

Baseline characteristics of pooled study populations were reported as median values and their interquartile ranges (IQR). In case the specific study adopted a matching approach to control confounding, the post-matching cohorts were included. Given the observational nature of the included studies, a random effect model was adopted in order to take into account the possible heterogeneity across the population. HR meta-analysis was performed after logarithmic transformation. We did not investigate publication bias due to the low number of studies included (<5). Heterogeneity across studies was assessed using the Cochran Q test. Higgins I^2^ statistics were used to determine the degree of between-study heterogeneity (I^2^ < 25%—low, 25–50%—moderate, >50%—high degree of heterogeneity).

The absolute risk reduction (ARR) was calculated by multiplying the meta-analytic HR (with the corresponding CI) by the assumed control risk (ACR) obtained from the meta-analysis of the incidence rate of the outcome (expressed as events/person-years) in non-AFCA cohorts. The number needed to treat (NNT) was normalized to the meta-analytic median follow-up.

*p*-values less than 0.05 were considered statistically significant. Statistical analyses were performed with R version 4.0.0 (R Foundation for Statistical Computing, Vienna, Austria).

## 3. Results

**Figure 1:**
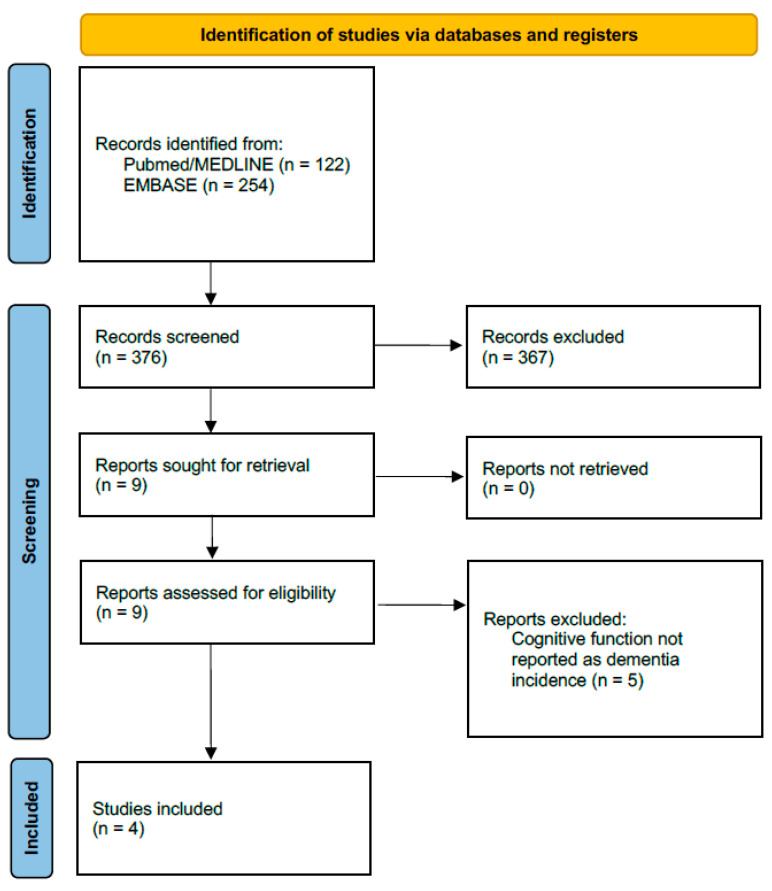
PRISMA flowchart.

The initial search retrieved 376 studies. After thorough evaluation, nine studies were assessed for eligibility, and four observational studies were finally included. Detailed descriptions of the selection process and data extraction are reported in the Supplementary Material. The final study population included 40,146 patients (11,312 in the AFCA cohort, 28,834 in the non-AFCA cohort) followed over a 4.5-year median follow-up (IQR 4.3–5.8 years). Figure 1 reports the PRISMA flowchart of the selection process. Table 1 summarizes the main characteristics of the included studies along with the study-specific statistical method to control confounding. Risk of bias evaluation using the Newcastle–Ottawa Scale is reported in Supplementary Table S2.

Baseline characteristics of the included populations are summarized in Table 2. Median age was 62.9 years (IQR 58.6–67.7 years), with nearly a 2:1 male-to-female ratio (males 65.4%, IQR 50.1–71.2%). The most common comorbidity was hypertension (62.0%, IQR 43.6–78.9%), whereas heart failure (30.3%, IQR 20.2–36.9%) and coronary/ischemic heart disease (8.8%, IQR 5.7–17.6%) affected a lower percentage of patients. A median of 13.3% (IQR 9.2–19.9%) patients had history of previous stroke and/or transient ischemic attack. The median CHA_2_DS_2_-VASc score was 2 (IQR 1.3–3.3), with 47.0% (IQR 41.7–55.9%) of patients on oral anticoagulants (OAC). Diagnosis of dementia was based in all four studies on International Classification of Diseases (ICD) codes (Table 1).

**Figure 2:**
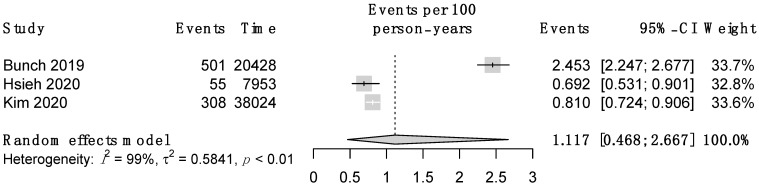
Forest plot of incidence rate of dementia in non-AFCA cohort.

**Figure 3:**
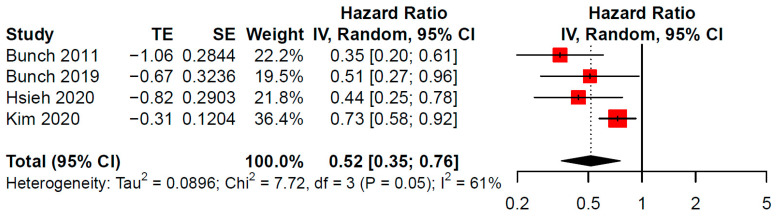
Forest plot for dementia occurrence comparing AFCA vs. non-AFCA cohorts.

The meta-analytic incidence rate of dementia was 1.12 events per 100 person-year (95% CI 0.47–2.67) (Figure 2). AFCA significantly reduced the incidence of dementia if compared to medical management (HR 0.52; 95% CI 0.35–0.76) (Figure 3). Based on these meta-analytic estimates, yearly absolute risk reduction was of 0.54 events per 100 person-year. Thus, the resulting NNT projected to the median follow-up (4.5 years) was of 41 AFCA procedures to avoid occurrence of dementia in one patient.

## 4. Discussion

Based on the present meta-analysis, AF catheter ablation halves the risk of dementia (HR 0.52, 95% CI 0.35–0.76) over a long-term follow-up (4.5 years). Assuming an event rate of dementia in non-AFCA patients of 1.12 events per 100 person-year, projected to the median follow-up duration, 41 procedures are needed to prevent one diagnosis of dementia.

Previous literature has shown that AF confers an increased risk of cognitive impairment and dementia. Since AF is a known prothrombotic state determining an increased risk of stroke, a potential genesis of cognitive decline due to incident cerebrovascular accident (CVA) is likely. However, the classical CVA-related component of dementia in AF patients might be less important than other quotes. In fact, AF has been associated with a 30% increase in dementia, independent of clinical CVA [23], supporting the hypothesis that AF might directly be linked to dementia. Several mechanisms may be involved: first, AF induces electrical and structural atrial remodeling, which further sustain AF and worsen the underlying atrial disease, increasing per se the risk of stroke and vascular dementia [24]. In addition, AF exerts detrimental hemodynamic effects on the cerebral blood flow, source of non-embolic subclinical cerebral ischemia and microbleeds, whose presence and frequency are associated with cognitive performance [7,8]. Overall, AF patients present decreased mean cerebral blood flow, especially in case of persistent AF, leading to reduced cerebral volume [25]. A computational model of cerebral circulation during AF also demonstrated that the irregularity in cardiac cycles led to transient episodes of hypoperfusion and hypertension in the deep cerebral circle [5,6]. This finding was recently confirmed in vivo by spatially resolved cerebral near-infrared spectroscopy (SRS-NIRS): interestingly, restoration of sinus rhythm by cardioversion related to a reduction in the abnormal cerebral hemodynamic events and normalization of brain tissue perfusion [26]. In this respect, the Acute Cognitive Changes During Atrial Fibrillation Episodes (AFCOG) study will evaluate the acute effect of an AF episode on cognitive function (NCT04033510) in patients managed by rhythm control strategy [27].

Although attention on this association has grown, definite evidence to determine the impact of AFCA and rhythm control strategies on cognitive function is lacking. The present analysis, despite using general terms to maximize sensitivity, retrieved a low number of studies. In fact, the AF-SCREEN International Collaboration has recently [14] highlighted the urge for dedicated research to establish the role of AF treatment strategies in preventing dementia. The effect of sinus rhythm maintenance has been investigated especially in regard to hard clinical endpoints, whereas evidence on cognitive impairment is limited and burdened by heterogeneity in the scale adopted to measure cognitive function. This inconsistency is reflected by the fact that current guidelines do not provide strong recommendations on assessing cognitive function, and only vaguely suggest the use of the Montreal Cognitive Assessment (MoCA) score to evaluate patient-reported outcomes [2]. On the other hand, the International Consortium for Health Outcomes Measurement does suggest to evaluate cognitive function by the use of a specific score (PROMIS) [28], on top of the known Mini Mental State Examination (MMSE) and/or other tools [29].

In any case, it is reasonable to assume that correct management of the arrhythmia holds the potential to delay or avoid cognitive decline occurrence. Since the association of AF and dementia includes several potential mechanisms, different strategies are under investigation.

As previously underlined, CVAs likely play an important role in dementia onset, and oral anticoagulant (OAC) drugs, by reducing CVA incidence in AF patients, may similarly reduce dementia incidence. In fact, Kim and al. recently showed that AF patients taking OAC have a lower risk of dementia than AF patients not prescribed OAC, also after censoring for stroke [30], suggesting that the protective effect is likely extended to subclinical cerebrovascular events. These data are, however, limited by their observational nature. Ongoing clinical trials on different anticoagulation strategies will assess the occurrence of cognitive function as a primary or secondary outcome [31,32]. The Blinded Randomized Trial of Anticoagulation to Prevent Ischemic Stroke and Neurocognitive Impairment in Atrial Fibrillation (BRAIN AF) will investigate if the risk of stroke, transient ischemic attack or cognitive decline is lowered in patients with non-valvular AF and low risk of stroke by the use of rivaroxaban 15 mg [32]. The Cognitive Decline and Dementia in Patients with Nonvalvular Atrial Fibrillation (CAF) Trial, instead, will investigate if AF patients randomized to dabigatran etexilate will have long-term higher cognition scores and lower rates of dementia compared to dose-adjusted warfarin [31]. The Trial of Apixaban vs. Warfarin in Reducing Rate of Cognitive Decline, Silent Cerebral Infarcts and Cerebral Microbleeds in Patients With Atrial Fibrillation (ARISTA), eventually, will assess whether anticoagulation with apixaban reduces the rate of decline in cognitive function when compared to warfarin, by reducing the rate of new cerebral infarction and cerebral microbleeds at cerebral MRI [33]. Even left atrial appendage closure will be investigated to prevent cognitive decline by the PLUG dementia trial [34].

On the other hand, the LOOP Study has shown that anticoagulation started after invasive AF screening did not reduce hard cardiovascular outcomes, potentially suggesting that not all AF patients detected on a screening basis might warrant anticoagulation or that this approach may not be effective alone [35]. In any case, although the STROKE-STOP trial failed to prove an impact of screening on dementia prevention [36], the effects of treating patients at an even earlier stage, identified by atrial high-rate episodes [37,38] or subclinical and asymptomatic AF [39,40,41], are currently under investigation.

Within all investigations, however, rhythm control appears as a promising manner to reduce dementia incidence [19]. Awaiting properly designed clinical trials, given that AFCA is the most effective approach to maintain long-term sinus rhythm, it appears rational to assess whether it might improve long-term cognitive function of AF patients. The CABANA trial [42] showed that AFCA was associated with a general benefit on quality of life, as well as with an increase in the mental domain of the SF-36 questionnaire; similarly, a recent study demonstrated MoCA score improvement in AFCA patients compared to those medically treated [18]. On the contrary, keeping in consideration that less than 20% of patients underwent AFCA, the EAST-AFNET 4 trial did not report differences [43] in cognitive function. Although more patients in the early rhythm control group maintained sinus rhythm at the end of follow-up, the cognitive performance scores were not significantly improved compared to the rate control group. The discrepancy between the EAST-AFNET 4 trial and present meta-analysis likely suggests that an even stricter rhythm control, achieved via AFCA, might be needed to improve cognitive function, or that the role of AFCA in dementia prevention might involve more aspects than sinus rhythm maintenance alone (e.g., improved atrial remodeling). The currently ongoing Cognitive Impairment in Atrial Fibrillation DIAL-F study (NCT01816308) will hopefully shed further light on the topic by comparing the effect of AFCA and AADs on cognitive impairment evaluated by MoCA score [44].

Eventually, considering the economic burden of dementia (about USD 200 billion in 2010 in the USA [45]) and the relatively small NNT to reduce dementia incidence found in this study, AFCA appears to be not only clinically effective in preventing a subtle complication of a disease with enormous social costs, but also to potentially reduce the associated healthcare economic burden.

### Limitations

The analysis is limited by the small amount of observational studies available on the topic and the inherent limitations of a meta-analysis. In particular, heterogeneity of study participants, as well as the lack of a standardized definition of dementia may partly affect the results. Moreover, even though matching and multivariable analysis control known confounders, residual bias attributable to unmeasured factors remains a concern. Due to the lack of arrhythmia details (e.g., type of AF, paroxysmal vs. persistent; arrhythmia duration) we could not perform subgroup analyses. In addition, the evaluation of dementia risk stratified by anticoagulation status has not been possible due to lack of the specific data in the original studies. Finally, due to the circumscribed baseline characteristics and outcome stratification, sensitivity analyses to assess the robustness of the results or to investigate any source of heterogeneity have not been performed.

## 5. Conclusions

The present analysis is, to the best of our knowledge, the first aiming to summarize currently available evidence on the potential role of AFCA in reducing cognitive burden. Based on the present meta-analytical results, AFCA relates to a nearly 50% reduction in incident dementia. However, the small number and observational nature of the retrieved studies underscore the need for high-quality randomized evidence on this epidemiologically and clinically extremely relevant topic.

## Figures and Tables

**Table 1:** General characteristics of the included studies.

Study	Database Description	Patients (Ablation Group)	Patients (Non-Ablation Group)	Follow-Up, Years	Dementia Diagnosis Definition	Statistical Analysis to Control Confounding
Bunch 2011 [21]	Intermountain Atrial Fibrillation Study Research Database	4212	16,848	4.1	ICD-9	Matching based on age and gender; multivariable Cox regression analysis
Bunch 2020 [22]	Intermountain Healthcare Registry	450	5336	5	ICD-9 and ICD-10	Matching based on age, gender and comorbidities *; multivariable Cox regression analysis
Hsieh 2020 [15]	Monocentric cohort and control group from National Health Insurance Research Database of Taiwan	787	787	9	ICD-9	Propensity score matching; multivariable Cox regression analysis
Kim 2020 [16]	Korean National Health Insurance Service Database	5863	5863	4.3	ICD-10 and use of dementia drugs	Propensity score matching, matching for health care utilization during follow-up and residential area, multivariable analysis

* Hypertension, coronary artery disease, diabetes and prior stroke.

**Table 2:** Patients’ baseline characteristics.

Study	Group	Male Gender (%)	Age (Years)	Hypertension (%)	CHF (%)	CAD (%)	Stroke/TIA (%)	Anticoagulant (%)	CHA2DS2-VASc Score (Median)
Hsieh 2020	AFCA	70.1	54.1	36.8	6.5	4.8	8.4	37.0	1.0
Hsieh 2020	Non-AFCA	70.0	54.9	36.8	6.4	2.1	3.6	56.9	0.0
Bunch 2011	AFCA	60.8	64.8	47.8	29.5	6.4	9.1 *		
Bunch 2011	Non-AFCA	60.8	66.0	45.3	23.6	6.4	10.5 *		
Kim 2020	AFCA	74.1	60.0	80.4	35.2	11.4	30.3 †	64.8 ǂ	2.0
Kim 2020	Non-AFCA	74.8	60.0	81.1	36.4	11.1	30.3 †	64.7 ǂ	2.0
Bunch 2020	AFCA	24.2	73.7	74.7	32.0	37.1	16.4	56.0	4.5
Bunch 2020	Non-AFCA	17.3	73.5	78.6	40.8	36.7	16.9	34.7	4.5

* The stroke/TIA percentage represents the addition of the individual stroke and TIA percentages reported in the original article. † The stroke/TIA percentage represents the addition of the individual hemorrhagic stroke, ischemic stroke and TIA percentages reported in the original article. ǂ The anticoagulant percentage represents the addition of the individual NOAC and Warfarin percentages reported in the original article.

## Data Availability

This review and its protocol were not registered in any database. Data supporting this article will be available under reasonable request to the corresponding author.

## Supplementary Materials

The following supporting information can be downloaded at: https://www.mdpi.com/article/10.3390/jcdd9050140/s1, Study selection; Data extraction; Table S1: PRISMA 2020 checklist; Table S2: Newcastle-Ottawa quality assessment scale table for cohort studies. [17,18,21,46,47,48] are cited in the Supplementary Materials.
