# Supplementary material for: Association of Catheter Ablation and Reduced Incidence of Dementia among Patients with Atrial Fibrillation during Long-Term Follow-Up: A Systematic Review and Meta-Analysis of Observational Studies
Source: J Cardiovasc Dev Dis. 2022 Apr 30;9(5):140. doi: 10.3390/jcdd9050140 (PMC9143892; doi:10.3390/jcdd9050140)
Supplement: Supplementary file 1 [file jcdd-09-00140-s001.zip › jcdd-1679103-supplementary.pdf]

Association of catheter ablation and reduced incidence of dementia among patients with atrial fibrillation during long-term follow-up: a systematic review and meta-analysis of observational studies

Supplementary Material

Study selection

We here provide a detailed description of the study selection process. 376 records were identified from database search. 367 were excluded through screening of title and abstract. 9 studies were retrieved and assessed for eligibility. Of these, 5 studies were excluded because they did not report data on *de novo* dementia occurrence; in particular:

- 1 study[1] compared cognitive function in patients undergoing radiofrequency and cryoballoon ablation using the Telephone Interview for Cognitive Status-modified (TICS-m) scale
- 3 studies[2–4] evaluated cognitive function through different scores as the Montreal Cognitive Assessment score (MoCA)
- 1 study[5] evaluated the effect of AFCA on cognitive function through neuropsychological testing at 24h before, 48h and 6 months after the index procedure.

Data extraction

Data on dementia incidence and baseline characteristics of the study population were extracted by evaluation of the published manuscript and supporting material. For 1 study[6], 95% confidence interval had been estimated by the available data since not expressively reported: the reported p-value was < 0.001, thus we consider the worst-case scenario were the p-value would be equal to 0.001 and calculated the corresponding confidence interval around the study-specific point estimate (to act more conservative as possible).

Supplementary references

1. Wang X, Wang Z, Yan X, Huang M, Wu Y. Radiofrequency and cryoballoon ablation improve cognitive function in patients with atrial fibrillation. *Medicine (Baltimore)* Medicine (Baltimore); 2021;**100**:e26914.

2. Tischer TS, Nitschke D, Krause I, Kundt G, Öner A, D'Ancona G, *et al.* Prevalence and Progression of Cognitive Impairment in Atrial Fibrillation Patients after Treatment with Catheter Ablation or Drug Therapy. *Cardiol Res Pract* Cardiol Res Pract; 2019;**2019**.

3. Piccini JP, Todd DM, Massaro T, Lougee A, Haeusler KG, Blank B, *et al.* Changes in quality of life, cognition and functional status following catheter ablation of atrial fibrillation. *Heart* Heart; 2020;**106**:1919–26.

4. Jin MN, Kim TH, Kang KW, Yu HT, Uhm JS, Joung B, *et al.* Atrial Fibrillation Catheter Ablation Improves 1-Year Follow-Up Cognitive Function, Especially in Patients With Impaired Cognitive Function. *Circ Arrhythm Electrophysiol* Circ Arrhythm Electrophysiol; 2019;**12**.

5. Zhang J, Xia SJ, Du X, Jiang C, Lai YW, Wang YF, *et al.* Incidence and risk factors of post-operative cognitive decline after ablation for atrial fibrillation. *BMC Cardiovasc Disord* BMC Cardiovasc Disord; 2021;**21**.

6. Bunch TJ, Crandall BG, Weiss JP, May HT, Bair TL, Osborn JS, *et al.* Patients treated with catheter ablation for atrial fibrillation have long-term rates of death, stroke, and dementia similar to patients without atrial fibrillation. J Cardiovasc Electrophysiol; 2011;**22**:839–45.

Table S1 PRISMA 2020 checklist

Section and Topic	Item #	Checklist item	Location where item is reported
**TITLE**			
Title	1	Identify the report as a systematic review.	Main Title
**ABSTRACT**			
Abstract	2	See the PRISMA 2020 for Abstracts checklist.	See the table below
**INTRODUCTION**			
Rationale	3	Describe the rationale for the review in the context of existing knowledge.	Introduction, Lines 44-49
Objectives	4	Provide an explicit statement of the objective(s) or question(s) the review addresses.	Introduction, Lines 50-53
**METHODS**			
Eligibility criteria	5	Specify the inclusion and exclusion criteria for the review and how studies were grouped for the syntheses.	Methods, Search strategy and primary outcome, Lines 61-69
Information sources	6	Specify all databases, registers, websites, organisations, reference lists and other sources searched or consulted to identify studies. Specify the date when each source was last searched or consulted.	Methods, Search strategy and primary outcome, Lines 57-58
Search strategy	7	Present the full search strategies for all databases, registers and websites, including any filters and limits used.	Methods, Search strategy and primary outcome, Lines 58-60
Selection process	8	Specify the methods used to decide whether a study met the inclusion criteria of the review, including how many reviewers screened each record and each report retrieved, whether they worked independently, and if applicable, details of automation tools used in the process.	Methods, Search strategy and primary outcome, Lines 69-73
Data collection process	9	Specify the methods used to collect data from reports, including how many reviewers collected data from each report, whether they worked independently, any processes for obtaining or confirming data from study investigators, and if applicable, details of automation tools used in the process.	Methods, Search strategy and primary outcome, Lines 69-73

Section and Topic	Item #	Checklist item	Location where item is reported
Data items	10a	List and define all outcomes for which data were sought. Specify whether all results that were compatible with each outcome domain in each study were sought (e.g. for all measures, time points, analyses), and if not, the methods used to decide which results to collect.	Methods, Search strategy and primary outcome, Lines 74-76
	10b	List and define all other variables for which data were sought (e.g. participant and intervention characteristics, funding sources). Describe any assumptions made about any missing or unclear information.	Methods, Search strategy and primary outcome, Lines 71-72, and Supplementary Materials, Data extraction section
Study risk of bias assessment	11	Specify the methods used to assess risk of bias in the included studies, including details of the tool(s) used, how many reviewers assessed each study and whether they worked independently, and if applicable, details of automation tools used in the process.	Methods, Search strategy and primary outcome, Lines 69-74
Effect measures	12	Specify for each outcome the effect measure(s) (e.g. risk ratio, mean difference) used in the synthesis or presentation of results.	Methods, Search strategy and primary outcome, Lines 66-67 and Supplementary Materials, Data extraction section
Synthesis methods	13a	Describe the processes used to decide which studies were eligible for each synthesis (e.g. tabulating the study intervention characteristics and comparing against the planned groups for each synthesis (item #5)).	Supplementary Materials, Data extraction section
	13b	Describe any methods required to prepare the data for presentation or synthesis, such as handling of missing summary statistics, or data conversions.	Supplementary Materials, Data extraction section
	13c	Describe any methods used to tabulate or visually display results of individual studies and syntheses.	Results, Figures 2-3
	13d	Describe any methods used to synthesize results and provide a rationale for the choice(s). If meta-analysis was performed, describe the model(s), method(s)	Methods,

Section and Topic	Item #	Checklist item	Location where item is reported
		to identify the presence and extent of statistical heterogeneity, and software package(s) used.	Statistical analysis, Lines 78-92
	13e	Describe any methods used to explore possible causes of heterogeneity among study results (e.g. subgroup analysis, meta-regression).	Methods, Statistical analysis, Lines 80-82 and Discussion, Limitations, Lines 240-242
	13f	Describe any sensitivity analyses conducted to assess robustness of the synthesized results.	Discussion, Limitations, Lines 240-242
Reporting bias assessment	14	Describe any methods used to assess risk of bias due to missing results in a synthesis (arising from reporting biases).	Supplementary Materials, Data extraction section
Certainty assessment	15	Describe any methods used to assess certainty (or confidence) in the body of evidence for an outcome.	Discussion, Limitations section
**RESULTS**			
Study selection	16a	Describe the results of the search and selection process, from the number of records identified in the search to the number of studies included in the review, ideally using a flow diagram.	Results, Lines 97-99 and Figure 1
	16b	Cite studies that might appear to meet the inclusion criteria, but which were excluded, and explain why they were excluded.	Supplementary Material, Study Selection section
Study characteristics	17	Cite each included study and present its characteristics.	Table 1, page 4
Risk of bias in studies	18	Present assessments of risk of bias for each included study.	Supplementary Material Table S2
Results of individual studies	19	For all outcomes, present, for each study: (a) summary statistics for each group (where appropriate) and (b) an effect estimate and its precision (e.g. confidence/credible interval), ideally using structured tables or plots.	Result, Figure 3, page 4
Results of syntheses	20a	For each synthesis, briefly summarise the characteristics and risk of bias among contributing studies.	Discussion, Limitations, Lines 233-238

Section and Topic	Item #	Checklist item	Location where item is reported
	20b	Present results of all statistical syntheses conducted. If meta-analysis was done, present for each the summary estimate and its precision (e.g. confidence/credible interval) and measures of statistical heterogeneity. If comparing groups, describe the direction of the effect.	Result, Figure 2-3, page 4
	20c	Present results of all investigations of possible causes of heterogeneity among study results.	Discussion, Limitations, Lines 240-242
	20d	Present results of all sensitivity analyses conducted to assess the robustness of the synthesized results.	Discussion, Limitations, Lines 239-241
Reporting biases	21	Present assessments of risk of bias due to missing results (arising from reporting biases) for each synthesis assessed.	Discussion, Limitations, Lines 233-238 and Supplementary Materials, Data extraction section
Certainty of evidence	22	Present assessments of certainty (or confidence) in the body of evidence for each outcome assessed.	Discussion, Limitations, Lines 240-242
**DISCUSSION**			
Discussion	23a	Provide a general interpretation of the results in the context of other evidence.	Discussion, Lines 208-230
	23b	Discuss any limitations of the evidence included in the review.	Discussion, Limitations section, Conclusion, Lines 248-250
	23c	Discuss any limitations of the review processes used.	Discussion, Limitations section
	23d	Discuss implications of the results for practice, policy, and future research.	Conclusion section
**OTHER INFORMATION**			
Registration and protocol	24a	Provide registration information for the review, including register name and registration number, or state that the review was not registered.	Registration information statement
	24b	Indicate where the review protocol can be accessed, or state that a protocol was not prepared.	Registration information

Section and Topic	Item #	Checklist item	Location where item is reported
			statement
	24c	Describe and explain any amendments to information provided at registration or in the protocol.	NA
Support	25	Describe sources of financial or non-financial support for the review, and the role of the funders or sponsors in the review.	Funding statement
Competing interests	26	Declare any competing interests of review authors.	Conflicts of Interests section
Availability of data, code and other materials	27	Report which of the following are publicly available and where they can be found: template data collection forms; data extracted from included studies; data used for all analyses; analytic code; any other materials used in the review.	Data availability statement

Abstract section

Section and Topic	Item #	Checklist item	Reported (Yes/No)
**TITLE**			
Title	1	Identify the report as a systematic review.	Yes
**BACKGROUND**			
Objectives	2	Provide an explicit statement of the main objective(s) or question(s) the review addresses.	Yes
**METHODS**			
Eligibility criteria	3	Specify the inclusion and exclusion criteria for the review.	Yes
Information sources	4	Specify the information sources (e.g. databases, registers) used to identify studies and the date when each was last searched.	Yes
Risk of bias	5	Specify the methods used to assess risk of bias in the included studies.	No
Synthesis of results	6	Specify the methods used to present and synthesise results.	Yes
**RESULTS**			
Included studies	7	Give the total number of included studies and participants and summarise relevant characteristics of studies.	Yes
Synthesis of results	8	Present results for main outcomes, preferably indicating the number of included studies and participants for each. If meta-analysis was done, report the summary estimate and confidence/credible interval. If comparing groups, indicate the direction of the effect (i.e. which group is favoured).	Yes
**DISCUSSION**			
Limitations of evidence	9	Provide a brief summary of the limitations of the evidence included in the review (e.g. study risk of bias, inconsistency and imprecision).	No

Section and Topic	Item #	Checklist item	Reported (Yes/No)
Interpretation	10	Provide a general interpretation of the results and important implications.	Yes
**OTHER**			
Funding	11	Specify the primary source of funding for the review.	NA
Registration	12	Provide the register name and registration number.	NA

Modified from: Page MJ, McKenzie JE, Bossuyt PM, Boutron I, Hoffmann TC, Mulrow CD, et al. The PRISMA 2020 statement: an updated guideline for reporting systematic reviews. BMJ 2021;372:n71. doi: 10.1136/bmj.n71

NA: Not applicable

Table S2. Newcastle-Ottawa quality assessment scale table for cohort studies.

Study	Selection	Comparability	Outcomes
Bunch et al. 2011	+++	+	++
Kim et al. 2020	++++	++	++
Bunch et al. 2020	+++	++	+
Hsieh et al. 2020	+++	++	+++
